# Induction of beige‐like adipocyte markers and functions in 3T3‐L1 cells by Clk1 and PKCβII inhibitory molecules

**DOI:** 10.1111/jcmm.17345

**Published:** 2022-07-08

**Authors:** Achintya Patel, Tradd Dobbins, Xiaoyuan Kong, Rehka Patel, Gay Carter, Linette Harding, Robert P. Sparks, Niketa A. Patel, Denise R. Cooper

**Affiliations:** ^1^ Department of Molecular Medicine University of South Florida Morsani College of Medicine Tampa Florida USA; ^2^ J.A. Haley Research Service Tampa Florida USA; ^3^ 7831 Department of Chemistry University of South Florida Tampa Florida USA

**Keywords:** adipocytes, Clk1, lipid droplets, mitochondria, PGC1α, PKCβII, UCP1

## Abstract

Excessive dietary intake of fat results in its storage in white adipose tissue (WAT). Energy expenditure through lipid oxidation occurs in brown adipose tissue (BAT). Certain WAT depots can undergo a change termed beiging where markers that BAT express are induced. Little is known about signalling pathways inducing beiging. Here, inhibition of a signalling pathway regulating alternative pre‐mRNA splicing is involved in adipocyte beiging. Clk1/2/4 kinases regulate splicing by phosphorylating factors that process pre‐mRNA. Clk1 inhibition by TG003 results in beige‐like adipocytes highly expressing PGC1α and UCP1. SiRNA for Clk1, 2 and 4, demonstrated that Clk1 depletion increased UCP1 and PGC1α expression, whereas Clk2/4 siRNA did not. TG003‐treated adipocytes contained fewer lipid droplets, are smaller, and contain more mitochondria, resulting in proton leak increases. Additionally, inhibition of PKCβII activity, a splice variant regulated by Clk1, increased beiging. PGC1α is a substrate for both Clk1 and PKCβII kinases, and we surmised that inhibition of PGC1α phosphorylation resulted in beiging of adipocytes. We show that TG003 binds Clk1 more than Clk2/4 through direct binding, and PGC1α binds to Clk1 at a site close to TG003. Furthermore, we show that TG003 is highly specific for Clk1 across hundreds of kinases in our activity screen. Hence, Clk1 inhibition becomes a target for induction of beige adipocytes.

## 1 INTRODUCTION

3T3‐L1 pre‐adipocytes are commonly used to study induction of beige‐like adipocytes from white adipose tissue. White and brown adipose tissue (WAT and BAT) are the two major types of mammalian fat. WAT specializes in the storage of excess energy in lipid droplets.^1^ BAT dissipates chemical energy in the form of heat. Interest in a third type of adipocyte, beige adipocytes, arises from the ability of circulating factors to increase energy expenditure in these cells by increasing expression of genes related to high mitochondrial content^2^ and elevated cellular respiration that is uncoupled from ATP synthesis.^3^ β‐adrenergic stimulation by epinephrine and norepinephrine increases lipid oxidation, and induces beiging of adipocytes. Norepinephrine, irisin, FGF21 and VEGF, in addition to other factors released by muscle and liver prevent obesity in high fat diet fed mice by inducing thermogenesis.^4^ VEGFR1 ablation induces tissue browning,^5^ and miR‐327 via FGF10‐FGFR2 can signal to promote browning.^6^ Cold temperatures also induce cells within white adipose tissue to form beige adipocytes that burn energy and generate heat. The signalling factors involved in both β‐adrenergic stimulation and cold exposure include transcriptional activators PPARγ, PGC‐1α and PRDM16, which together induce uncoupling protein‐1 (UCP1)‐containing mitochondria.^4^ Additionally, factors including bone morphogenic peptide, and circulating factors such as thyroid hormones, bile acids,^4^ natriuretic peptides, retinoids and various cytokines can regulate a thermogenic program in WAT.^7^

Other molecules such as salsalate, nitrate, peroxisome‐ proliferator‐activated receptor‐γ (PPARγ) agonists, adenosine and lactate induce thermogenic adipocytes.^8^ In these cases, beiging of WAT is dependent on maintaining the thermogenic response and retaining brown‐like thermogenically competent adipocytes.^9^ The identity of a signalling pathway to induce mitochondrial biogenesis and decrease lipid storage without β‐adrenergic stimulation has not been described.

Clk1, a kinase regulating alternative splicing of PKCβ pre‐mRNA for PKCβII, is highly regulated during adipogenesis. Differentiation to mature, lipid containing adipocytes is blocked by its inhibition of pre‐adipocytes.^10^, ^11^ This occurs via RNA splicing proteins containing serine/arginine (SR) rich domains. They determine splice sites in relevant pre‐mRNA during adipogenesis. PGC1α contains a SR (serine‐arginine) domain targeted by Clk. Clk2 phosphorylation results in disruption of a PGC1α/MED1 complex. PGC1α can also act as a splicing protein along with its transcriptional roles.^12^, ^13^ We hypothesize that based on the prediction of motifs via bioinformatics, Clk1 and PKCβII can phosphorylate PGC1α. It follows that Clk1 phosphorylation may ‘repress’ its activity in differentiating WAT. However, when Clk1 or PKCβII kinase activities are blocked/inhibited, un‐phosphorylated PGC1α is ‘permissive’, making it available for UCP1 transcription, which increases proton leak in mitochondria.

TG003 is an efficient inhibitory molecule of Clk1/2/4 kinases.^14^ Depending upon its concentration, TG003 blocks Clk1/4 (IC_50_ 15–20 nM) or Clk2 (IC_50_ > 200 nM) activity. In adipose tissue, Clk1 is highly expressed.^10^, ^15^ We previously identified the insulin/PI3Kinase/Akt/Clk1/PKCβII pathway for regulating glucose transport in adipocytes.^10^ In the present study, we tested whether inhibition of Clk1 or PKCβII activity was involved in beiging of adipocytes.^2^

TG003 was added to 3T3‐L1 cells stimulated to differentiate. On day 3 (D3), media contained only insulin and FBS. Differentiating cells were treated up to day 8 (D8) to determine effects on beiging factors. CG53353, a specific PKCβII inhibitor, was added similarly to determine its effects on beiging.

## 2 MATERIALS AND METHODS

### 2.1 Cell culture

Mouse 3T3‐L1 pre‐adipocytes (ATCC CL‐173™) were authenticated by their ability to differentiate to white adipocytes. Pre‐adipocytes were maintained prior to differentiation in DMEM high glucose (Invitrogen) with 10% bovine calf serum (Sigma‐Aldrich) at 37°C and 5% CO_2_. Confluent cells (95%) were differentiated on ‘Day 0’ in DMEM high glucose with 10% foetal bovine serum (FBS) (Atlas Biological), 10 μg/ml bovine insulin (Sigma‐Aldrich), 1 μM dexamthasone (Sigma‐Aldrich), a and 0.5 mM isobutyl‐1‐methylxanthine (Sigma‐Aldrich). On D3, media was replaced with DMEM high glucose, 10% FBS, bovine insulin (10 µg/ml), and inhibitors (TG003 [50 nM] or CGP53353 [10 µM]) (both from Sigma‐Aldrich). On D6, media was changed again to DMEM high glucose with 10% FBS and inhibitors were again added to cells. On D8, images of the cells were captured after staining with Oil Red O (Sigma‐Aldrich) or MitoTracker™ green FM (Invitrogen); cells were harvested for RNA extraction or protein lysates according to manufacturer's instructions, and were subsequently analysed for UCP1, PGC1α and PPARγ by microarray, RT‐PCR and Western blot.

### 2.2 Oil Red O staining for lipids and lipid droplets

3T3‐L1 adipocytes were washed with PBS and fixed with 10% formalin for 30 min, then rinsed and incubated with Oil Red O staining solution (Adipogenesis Assay kit, Millipore Sigma) for 5 min. After washing, images were captured with an Olympus 1 × 70 fluorescent microscope. For quantification, 500 μl of dye extraction solution was added to cells and incubated for 30 min. Absorbance was read at 485/490 nm and compared with control cells.

### 2.3 Western blot

3T3‐L1 cells were harvested in lysis buffer containing protease inhibitors (Sigma*Fast* Protease Inhibitor Tablet, Sigma‐Aldrich) and phosphatase inhibitors (Phosphatase Inhibitor Cocktail 1, Sigma‐Aldrich). Lysates were run on 10% SDS‐PAGE gels and transferred to Hybond‐C Extra nitrocellulose membranes (Amersham). Membranes were blocked and probed in 5% non‐fat dried milk. Detection was performed using SuperSignal West Pico Chemiluminescent substrate (Pierce Biotechnology). Antibodies were as follows: PPARγ (sc7273), PGC1α (sc518025), UCP1 (sc293418) and β‐actin (sc47778) from Santa Cruz Biotechnology. CIDEA (Millipore ABC350) antibody was from Sigma‐Aldrich.

### 2.4 RT‐PCR

RNA was extracted using RNAeasy Mini kit (Qiagen) and Reverse Transcriptase was performed using Omniscript RT kit (Qiagen, #205113) according to manufacturers' protocols. PCR reactions were performed on cDNA to determine mRNA expression by qPCR using primers purchased from Taqman by Applied Biosciences as follows: GAPDH (Mm99999915_gl), Clk1 (Mm00438254_m1), Clk2 (Mm00432578_ml), Clk4(Mm01288915_m1), UCP1 (Mm00494069_m1), PGC1α (mM01208835_m1) and PPARγ (Mm00440940_m1). The Mouse Adipogenesis RT^2^ profiler PCR Array (Qiagen) was performed on control and TG003‐treated 3T3‐L1 adipocytes (D8).

### 2.5 SiRNA transfection

SiRNAs that targeted separate areas of mRNA were pretested and the most efficient were used to deplete Clk1,2 and 4. Clk1 siRNAs (IDs: SR300856C), Clk2 siRNAs (IDs:SR416008A‐C) and Clk4 siRNAs (IDs: SR408785A‐C) along with scrambled control siRNA was purchased from Origene, and transfected using Origene's siTran transfection reagent. Cells were treated with the siRNA + Origene siTran transfection agent on Day 2 of differentiation. SiRNA was re‐transfected with every media change until the cells were harvested for RNA.

### 2.6 Mitochondrial abundance

Mitochondria were assessed using MitoTracker Green™FM dye (Invitrogen, MM7514) according to the manufacturer's instruction in live 3T3‐L1 adipocytes (D8).

### 2.7 Mitochondrial stress test

3T3‐L1 pre‐adipocytes were plated at 1000 cells per well in collagen coated microplates provided by Agilent for the Seahorse XFp Extracellular Flux Analyzer (Agilent Technologies). Cells were cultured in 100 µl differentiation media described above. D3 cells were treated with 50 nM TG003 until D8. TG003 was replaced every 2 days with media changes. Cells were then switched to CO_2_ and serum‐free media (Part #103575‐100) containing 10 mm glucose, 2 mM L‐glutamine and 1 mM sodium pyruvate and the sensor cartridge ports were 1.0 µm Oligomycin (Port A), 1 µm FCCP (Port B) and 0.5 µM Rotenone/Antimycin A (Port C). Cells were preincubated for 1 h at 37°C without CO_2_ prior to the Mitochondrial Stress Test (Agilent Technologies) where readings of oxygen consumption rate (OCR) were recorded. The Stress Test report generator calculated the parameters using WAVE and data were exported to Excel. Results are from four different assays in triplicate using different passages of cells with similar results.

### 2.8 Computational modelling

Schrödinger's Maestro program (version 9.3.5) as graphical interface and Maestro version 10.2 (Schrödinger, LLC) was used for ligand interaction diagramming. Virtual screening was performed on TG003 prepared with Schrödinger's LigPrep program. TG003 was docked using Schrödinger's GLIDE software on Clk1 using a grid generated from the coordinates of the ligand bound in crystal structure. Characterization of protein–protein interaction between PGC1α (PDB: 5UNJ) (Novus Biologicals #H00010891‐Q01) and Clk1 used the default settings of web server ClusPro.^16^, ^17^ Using the minimized structure of Clk1 (PDB ID: 1Z57) (AbCam #114 706), molecular dynamics simulations were conducted with NAMD 2.12^18^ using the CHARMM36m force field.^19^ The system used the CHARMM‐GUI solution builder, with a concentration of 150 mM NaCl. Simulation parameters included constant pressure of 1 atm via Langevin dynamics, and a constant temperature of 310 K using Langevin piston Nosé−Hoover methods.^20^ Long‐range electrostatic forces were evaluated using the Particle Mesh Ewald (PME) with a 1 Å grid spacing.^21^, ^22^ Van der Waals interactions were calculated using a 12 Å cut‐off with a force‐based switching scheme after 10 Å, as well as a 2 fs time step integration via the SETTLE algorithm.^23^ The system equilibrated for 10 ns restraining the Cα atoms of the protein (1.0 kcal/mol/Å^2^) to allow for solvation. This was followed by a production run of 100 ns without restraints.^24^ Visualization and analysis used VMD 1.9.3 and RMSD calculated using the trajectory tool and displayed using Microsoft Excel.^25^

### 2.9 Surface plasmon resonance

Surface plasmon resonance (SPR) measurements were performed on a Biacore T200 instrument equipped with CM5 sensor chip with ~4000 response units (RU) Clk4 (Abcam#AB204144), ~400 RU Clk1 and ~14,000 RU Clk2 (Abcam #AB63190) covalently immobilized with NHS/EDC to the surface. Additionally, SPR was performed on ~300 RU Clk1, 1000 RU Clk2 and 1000 RU Clk4 covalently attached to a CM5 Chip. For Clk1, Ni‐NTA SPR was used non‐covalently attaching His‐Labeled Clk1 at ~1800 RU for Clk1 binding to TG003 and for TG003 inhibition of PGC1α to Clk1 approximately ~1000 RU of PGC1α was non‐covalently attached to the chip. TG003 and PGC1α were titrated and flowed over these chips in 10× HBS‐N buffer from GE diluted in ultrapure water and filtered. Binding was expressed in relative response units (RU); the difference in response between the immobilized protein flow cell and the corresponding control subtracting blanks and utilizing a solvent control for DMSO generated from 0.5, 0.75, 1, 1.25 and 1.5 percent DMSO. TG003 was applied to chips containing Clk1,2 and 4 using 1:1 titrations and results exported from BiaEvaluate software into GraphPad prism (GraphPad Software). Saturation curves for PGC1α binding to Clk1 were fit using a specific binding equation with Hill slope, whereas all other SPR saturation curves were fit using a 1:1 specific binding model.

### 2.10 Kinase activity assay

TG003 was tested for specificity using a panel of 370 kinases provided by Reaction Biology Corporation. TG003 was added at 50 nM and a control compound, Staurosporine. The reactions were carried out using 10 μM ATP by Reaction Biology.

### 2.11 Statistics

Results are expressed as the mean ± SEM, where n equals the number of independent experiments in which replicate analyses were performed. Significant differences were assessed using Student's *t*‐test with *p*‐values ≤0.05 being considered significant performed with ANOVA when appropriate using GraphPad Software.

## 3 RESULTS

### 3.1 Treatment of 3T3‐L1 cells with TG003 on D3 through D8, decreased lipid droplet size

3T3‐L1 pre‐adipocytes differentiated as described, showed that 90% of cells had efficiently differentiated to adipocytes, and accumulated large lipid droplets, which are considered white adipocytes by D8. The ability of 3T3‐L1 cells to differentiate to beige adipocytes was previously shown using capsaicin, which induced a ‘brite’ phenotype.^26^ Prolonged Rosiglitazone and T3 in addition to IBMX also increased beige‐like gene profiles in 3T3‐L1 adipocytes.^2^ T3 is frequently used during brown/beige adipocyte differentiation.^2^ Capsaicin and Resveratrol are compounds known to block alternative splicing of pre‐mRNA transcripts,^26^, ^27^, ^28^, ^29^ and they also induce beiging of adipocytes. So, this was our rationale for modulating alternative splicing during the transition of white to beige adipocytes by targeting Clk1/2/4 with TG003, the most efficient inhibitor of splicing.

**FIGURE 1:**
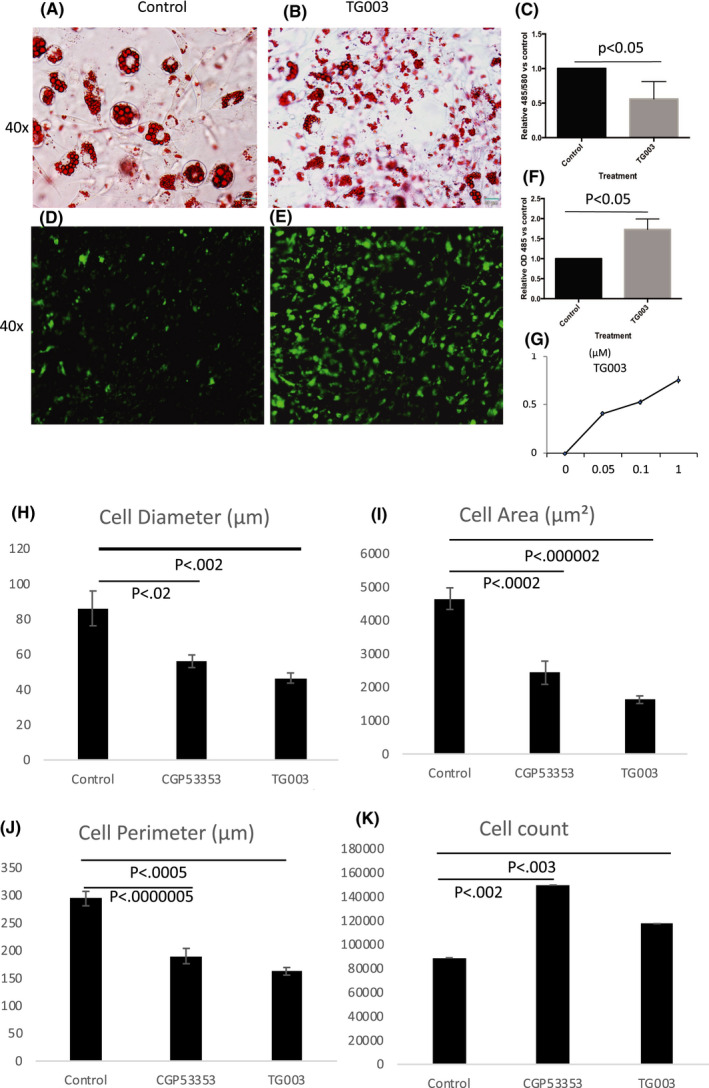
Oil Red O stain of lipid deposits in mature (Day 8) untreated 3T3‐L1 adipocytes (A) and mature 3T3‐L1 adipocytes treated with TG003 (50 nM) since Day 3 of differentiation (B). The lipid droplets in untreated cells are larger than those in TG003‐treated cells, with the ORO stain indicating the presence of lipids. (C) Graphical representation of the extracted lipid content of the control and TG003‐treated 3T3‐L1 cells. (D) Image of the mitochondrial number in control 3T3‐L1 adipocytes using MitoTracker green as described in Methods. (E) Image of mitochondria in cells as described in D but treated with TG003 (50nM) since Day 3. (F) There was twofold increase in cellular mitochondria with TG003 treatment as shown by the quantification of the extracted cellular dye. (G) Graphs the response to TG003 concentration between 50 nM and 1 µM when MitoTracker green dye concentrations were recorded after solubilization as described. Data for Oil Red O and MitoTracker green dye concentrations from 3 separate experiments performed in duplicate (C and F) were analysed using a two‐tailed statistical *t*‐test with *p* < 0.05 as indicated. Scale for 20 µm is shown in blue on A and B frames in the lower right‐hand corner. (H) The cell diameter, (I) cell area, (J) cell perimeter and (K) cell counts per area, were measured by an Olympic microscope CellSens software. The data shows mean ± SEM with significant differences (*p* < 0.01 to *p* < 0.000001) between cells treated with CGP53353 or TG003 compared to untreated cells in all parameters as indicated from 3 separate areas analysed of representative images

We treated 3T3‐L1 cells with 50 nM of TG003 on D3 of differentiation. This concentration was considered to be specific for Clk1/4 inhibition as higher levels (>200 nM) are required for Clk2. Figure 1A–C shows that 3T3‐L1 pre‐adipocytes treated with TG003, accumulated fewer lipids in droplets on D8. When Oil Red O stain was extracted from adipocytes, the accumulated stain was reduced by >50%. Figure 1H–K indicated that the average cell diameter was 45% less in TG003‐treated cells. The cell area was likewise reduced by up to threefold and this was also noted in the cell perimeter. Cell counts were higher in TG003‐treated cultures than in control cultures.

The biogenesis of new mitochondria is also an earmark of the beiging process. Mitochondria, central in metabolism of adipose tissue, contribute to lipolysis and lipogenesis.^30^ Brown and beige adipocytes, when activated by sympathetic stimulation, dissipate chemical energy, which is stored as triglycerides by channelling them into β‐oxidation,^31^ and this energy is converted into heat, as non‐shivering thermogenesis. Uncoupling proteins (UCP) are mitochondrial inner membrane proteins that cause inducible proton leak.^32^ There are five UCPs, but UCP1 is expressed at higher levels in brown adipocytes and is the best studied.^31^ Since beige adipocytes contain more mitochondria which contain more UCP1 than white adipocytes,^33^ we used MitoTracker dye uptake by living cells to visualize mitochondria. Mitochondrial content was greater in TG003‐treated cells than in control cells by twofold as shown in Figure 1D–F. Adipocytes that had been initially treated on D6 rather than D3 also demonstrated a TG003 response to increase mitochondrial content (data not shown). A dose response to TG003 showed that the maximal response was at 100 nM (Figure 1G).

### 3.2 Microarray analysis of markers associated with adipogenesis

**FIGURE 2:**
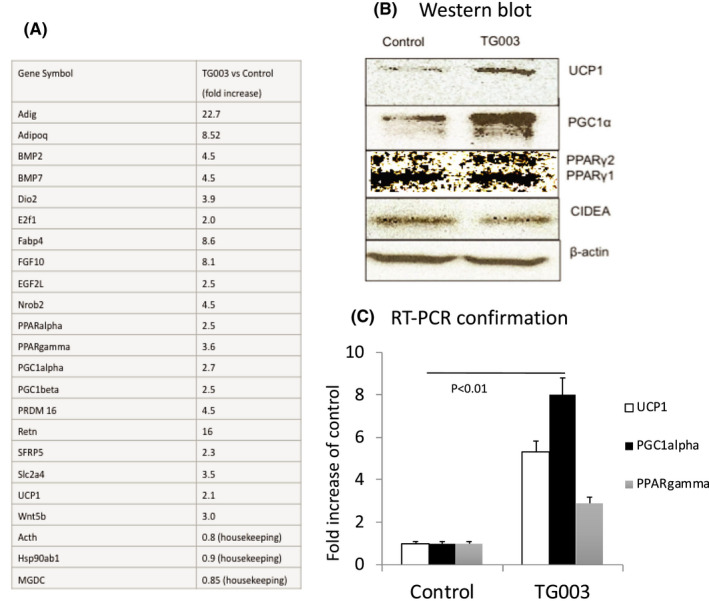
Induction of brown/beige adipocyte genes by TG003 (50 nM) treatment of 3T3‐L1 adipocytes. (A) PCR microarray for adipogenesis results indicate 2–22 fold increases in adipogenic and brown/beige adipocyte genes when untreated adipocytes are compared with TG003 (50 nM)‐treated 3T3‐L1 adipocytes. (B) Western blot confirmation of protein concentrations for UCP1, PGC1α and PPARγ1+2 (splice variants) in addition to CIDEA. (C) RT‐PCR confirmation of UCP1, PGC1α and PPARγ1 + 2 mRNA levels. Differences in levels via RT‐PCR versus the microarray reflect the potential difference in primers designed to amplify the target as well as the optimization of RT‐PCR vs the microarray. (*p* < 0.01 for each target vs. control)

The appearance of fewer lipid droplets and increased mitochondria with TG003 treatment suggested that 3T3‐L1 cells were morphologically and phenotypically altered with Clk1/4 inhibition. We used a PCR array (RT^2^ Profiler, Quiagen.com) to examine a panel of mouse adipogenesis RNA. We compared control with TG003 (50 nM)‐treated adipocytes as described above on D8. Figure 2A indicates multiple gene symbols predicting pro‐brown adipocytes with changes exceeding twofold expression vs. the control after treatment with TG003.

BMP7, Dio2, PPARγ, PGC1α, Prdm16, UCP1 and Wnt5b were increased by more than twofold. Of the pro‐brown genes expressed, we selected UCP1, PGC1α, PPARγ1 and 2 to confirm by PCR and Western blot.

### 3.3 TG003 treatment increased UCP1, PGC1α and PPARγ in differentiated 3T3‐L1 cells

UCP1 expression is higher in beige adipocytes than WAT in mammals,^34^ and UCP1 was not always detected in cells without β‐adrenergic activation.^35^ Figure 2B indicates that UCP1 protein levels were 10‐fold higher than in control adipocytes. UCP1 mRNA induction was only five‐fold greater. PGC1α protein levels were highly expressed (20‐fold higher) but the mRNA levels were only 2‐fold higher. This suggests that the protein levels are sustained and stabilized in these cells as reported by others.^36^

Although PPARγ1 levels were not elevated in Western blots, PPARγ2 levels were two‐fold higher in RT‐PCR confirming the microarray (Figure 2C).

### 3.4 Treatment of 3T3‐L1 cells with CGP53353, a PKCβII‐specific inhibitor, decreased lipid storage and increased mitogenesis

**FIGURE 3:**
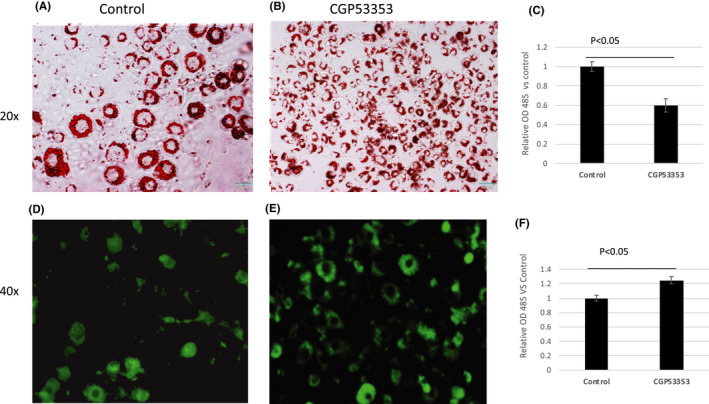
Cells treated with the specific PKCβII inhibitor, CGP53353, also demonstrated less lipid accumulation and more mitochondria. (A) Control vs (B) CGP53353 treatment of 3T3‐L1 adipocytes stained with ORO. (C) quantifying the decrease. (D) Control vs (E) CGP53533 treatment of cells stained with MitoTracker green and (F) quantifying the increase. (*p* < 0.05 as determined using a Student's *t*‐test from 3 separate analyses of extracted ORO or MitoTracker green). Scale of 20 µm is shown in blue in the lower right‐hand corner of frames A and B

PKCβII is a mediator of adipogenesis.^10^ Its absence in a global PKCβ knockout model results in mice that are resistant to diet‐induced obesity (DIO).^37^ Since insulin‐activated Clk1 mediates the splicing of PKCβII and its expression, we next evaluated the effect of a PKCβII kinase inhibitor, CGP53353. Treatment decreased Oil red O accumulation by 50% (Figure 3A–C). MitoTracker green uptake was increased >20% (Figure 3D–F). Additionally, CPG53353 decreased cell size and increased cell count as shown in Figure 1.

Inhibition of PKCβII splicing, and also the splicing of PPARγ2,^11^ are downstream events of Clk inhibition.^38^ PKC isozymes have phosphorylation sites on PGC1α in addition to Clk1 sites as determined by PhosphoMotif Finder on the Human Protein Reference Database. The role of PKCβII phosphorylation on PGC1α has not been described in beiging.

### 3.5 Clk1 siRNA increased UCP1 expression similar to TG003 treatment

**FIGURE 4:**
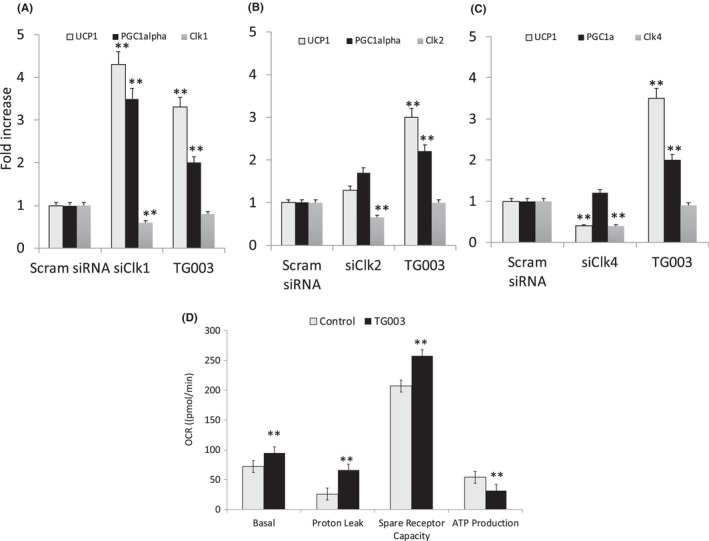
SiRNA reduction of Clk 1, 2 and 4 levels by specific siRNA reveals that Clk1 inhibition regulates levels of UCP1 and PGC1α. (A) siRNA to Clk1 reduced Clk1 levels by 50% while UCP1 mRNA levels increased fourfold, and PGC1α increased 3.5‐fold. (B) siRNA to Clk2 reduced Clk2 levels by 40%, but only increased UCP1 mRNA by 20% while PGC1α was only increased 50%. (C) siRNA to Clk4 reduced Clk4 by 70%; it reduced UCP1 mRNA by 70%, and PGC1α was increased only 15%. A–C. TG003 (50 nM) treatment consistently increased UCP1 by 3–4 fold, PGC1α by twofold, and had minimal effects on lowering Clk levels. (**p < 0.01 for siClk1,2 and 4 or TG003 vs. scrambled siRNA which was equivalent to control in all cases as evaluated using ANOVA). (D) Oxygen Consumption Rates (OCR) for untreated and TG003‐treated 3T3‐L1 adipocytes. Basal OCR increased 25 pmol/min in TG003‐treated cells vs. control. Proton leak was increased 2.3‐fold with TG003 treatment. Spare receptor capacity increased 50 pmol/min while ATP production was reduced approximately 45% in TG003‐treated cells. Data shown are mean ± SEM (**p < 0.01 for 5 separate experiments performed in duplicate and analysed by Friedman's paired *t*‐test)

Our previous studies indicated that an inactive Clk1 blocked adipogenesis in 3T3‐L1 pre‐adipocytes.^10^ Since TG003 inhibits Clk1, 2 and 4 depending upon its concentration, we investigated which isoform was involved with siRNA from Origene. Cells were treated with TG003 (50 nM) for comparison, and a scrambled siRNA mix was the control. Transfection of siRNA for Clk1 reduced Clk1 expression by fifty percent, and resulted in increased UCP1 and PGC1α mRNA as shown in cells treated with TG003 (Figure 4A). SiClk2 and siClk4 resulted^11^ in fifty percent reduction of the respective Clk mRNA levels but failed to significantly upregulate UCP1 or PGC1α mRNA to the level noted with TG003 (Figure 4B,C). Hence, Clk1 depletion specifically upregulated genes for beiging. It was noted that Clk4 depletion blocked UCP1 expression.

### 3.6 TG003‐treated adipocytes increased proton leak and decreased ATP production

The benchmark for functional UCP1 in beige adipocytes is the ability of these cells to demonstrate increased proton leak. The Seahorse XFp mitochondrial stress test determined proton leak following administration of oligomycin to control and TG003‐treated adipocytes. Figure 4D shows basal oxygen consumption rate (OCR) significantly higher in TG003‐treated cells. OCR for proton leak was up to threefold higher in TG003‐treated cells. OCR for spare receptor capacity was consistently higher in treated cells, and OCR for ATP production was significantly reduced in these cells. This change in function is consistent for beige adipocytes or rosiglitazone‐treated cells (results not shown).

### 3.7 Clk1 binds TG003 and PGC1α with higher affinity than Clk2, and TG003 and PGC1 bind at the same region of Clk1

**FIGURE 5:**
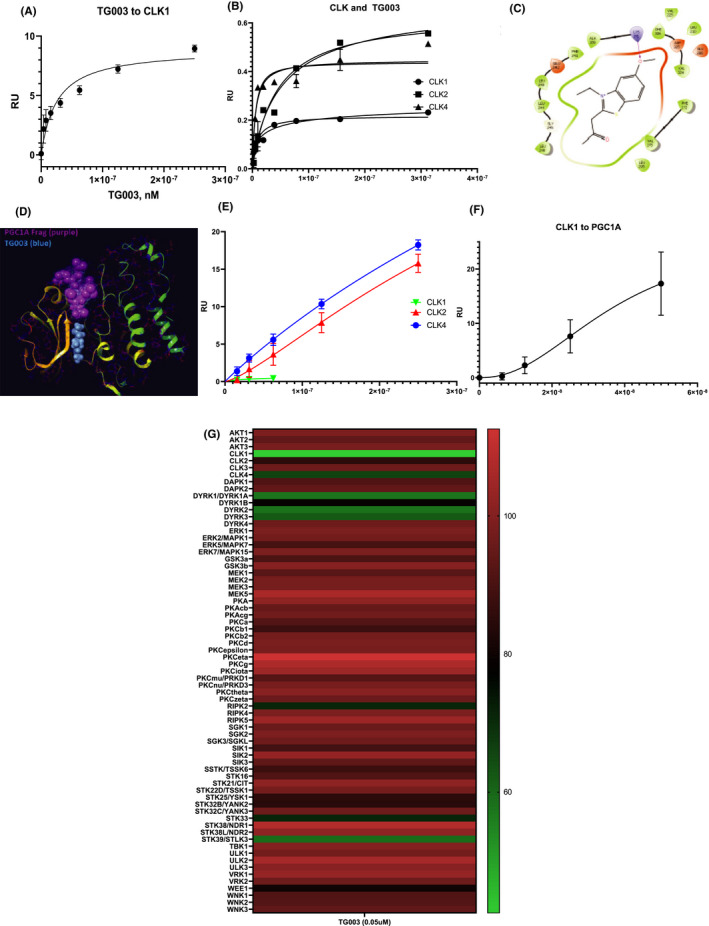
Binding of TG003 and PGC1α to Clk 1. (A) Steady state SPR of ~1800 RU Clk1 affixed to an Ni‐NTA chip and titrated with TG003 in HBS‐N with 1% DMSO plotted in GraphPad Prism using an one‐site specific binding model with a K_D_ of 25.24 with a 95% confidence interval of 12.93 to 48.53 nM. (B) Steady state SPR of ~400 RU Clk1, ~1000 RU of Clk4 and ~1000 RU Clk2 attached to a CM5 chip via NHS/EDC chemistry indicating binding of Clk1 and Clk4 to TG003 greater than binding of Clk2 to TG003. (C) Steady state SPR of Clk1, Clk2 and Clk4 as in 7B, indicating affinity of Clk1 to PGC1α of 240 nM +/− 28 nM, Clk2 to PGC1α of 980 nM +/− 89 nM, and Clk4 to PGC1α of 509 nM +/− 73 nM. (D) Ligand interaction diagram of TG003^5^ bound to Clk1 (PDB ID: 1Z57) generating grid from ligand in crystal structure. (E) 100 ns simulation of Clk1 in the absence of ligand prepared using Schrodinger Protein Preparation Wizard on PDB ID: 1Z57 and NAMD run using CHARMM‐GUI Solution Builder with RMSD of aligned backbone taken over the 1000 frames of the trajectory with each frame equal to 100 ps. (F) ClusPro predicted binding of PGC1α fragment from (PDB ID: 5UNJ) minimized with Protein Preparation Wizard and bound to receptor Clk1 from 7D aligned and one CLK deleted indicating proximal binding to nearby region of Clk1. (G) Heatmap to visualize inhibition of kinases by TG003

Our cellular studies investigated the functional aspects of TG003 treatment of adipocytes. To further clarify the role of Clk1, we performed studies using recombinant Clk1, 2 and 4, with TG003 and PGC1α. TG003 was reported in 2004 as a specific inhibitor that binds Clk1 and Clk4 preferentially over Clk2.^14^ TG003 and similar compounds were explored via crystallography to determine binding modes of TG003 for Clk1 (Figure 5D) (https://www.ncbi.nlm.nih.ov/pubmed/21276940). We non‐covalently chelated Clk1‐6Xhis construct to the surface of a Ni‐NTA chip with approximately 1800 RU Clk1 and titrated 1:1 TG003 in HBS‐N with 1% DMSO at 30 µl/min for 60 s association period followed by 120 s disassociation. Using the Biacore Evaluation software, the K_D_ was determined to be 25 nM +/− 1.3 nM for Clk1 using GraphPad Prism 8.4.3 one‐site specific binding model. (Figure 5A) as compared to the reported IC_50_ from Sigma‐Aldrich of 20 nM. We used these results to calibrate a CM5 chip crosslinked via NHS/EDC chemistry with a blank flow cell 1, flow cell with ~100 RU Clk1, and exactly 1000 RU Clk2 and 1000 RU Clk4 on flow cells 3 and 4, respectively. We simultaneously flowed TG003 on all flow cells (Figure 5B) and PGC1α (Figure 5E). K_D_'s were determined using this chemistry and same flow rate as in Figure 5A, reporting 95% confidence intervals (CI) with a one‐site specific binding model in GraphPad for Clk1 GST 8 nM (4–16 nM CI), Clk2 was 43 nM (25–73 nM CI) and Clk 4 was 6 nM (3.1 to 9.7 nM CI) in Figure 5B using GraphPad Prism 8.4.3 one‐site specific binding model, which is in line with previous reported K_D_'s^12^ and IC_50_'s reported. We hypothesized that TG003 may affect binding of PGC1α to Clk1 so we set out to determine the TG003 binding mode for Clk1 using crystal structure (PDB ID:1Z57) and docking TG003 using a receptor grid selecting the crystallized ligand as the centre of this box (https://www.ncbi.nlm.nih.gov/pubmed/19278650). The binding mode of the highest affinity pose using Schrodinger Glide SP and exported in a Ligand Interaction Diagram is displayed (Figure 5C).

To establish a relevant Clk‐binding portion of PGC1α taken from (PDB ID: 5UNJ), we used the ClusPro protein–protein docking software to determine potential binding regions of this fragment for Clk1 in Figure 5D (https://www.ncbi.nlm.nih.gov/pubmed/28363985). When superimposing the pose from Figure 5C onto the pose generated from the ClusPro server for Clk1 bound to PGC1α fragment, we see close proximity of these two molecules in the Clk1 active site indicating the potential for molecules reported that bind to this site, to potentially affect binding of PGC1α (https://www.ncbi.nlm.nih.gov/pubmed/28363985 *Id*.). These results indicate that TG003 is a suitable molecule to study the interaction of PGC1α with Clk1. We then titrated PGC1α in HBS‐N without DMSO at a flow rate of 30 µl/min for 60 s association and 120 s disassociation in Figure 5E indicating affinity of Clk1 to PGC1α of 240 nM +/− 28 nM, Clk2 to PGC1α of 980 nM +/− 89 nM, and Clk4 to PGC1α of 509 nM +/− 73 nM using GraphPad Prism 8.4.3 one‐site specific binding model. Notably, relative RU for Clk1 binding to PGC1α is lower using the CM5 chip due to a magnitude less Clk1 on the chip than Clk2 and Clk4. We carried out a similar experiment to Figure 5A to generate an accurate K_D_ of Clk1 binding to PGC1α for the same reasoning of not being able to affix enough Clk1 to the CM5 chip. Utilizing a PGC1α 6X his‐tagged construct we affixed 1000 RU of PGC1α to an Ni‐NTA chip and using same flow rates as in Figure 5E, we determined the affinity of Clk1 to PGC1α to be similar to that reported in Figure 5E with a K_D_ of ~38 nM using GraphPad Prism 8.4.3 one‐site specific binding model with Hill slope.

### 3.8 Clk1 Inhibition of binding to PGC1α and kinase panel

To further establish if beiging resulting from TG003 inhibition was specific to Clk1, we first determined whether TG003 inhibited PGC1α binding to Clk1, Clk2 and Clk4 using SPR. We used the same chip for Clk2 and Clk4 as was used in Figure 5B,C at a concentration of 500 nM TG003 and determined that there was roughly 10% inhibition of Clk2 binding to PGC1α and roughly 40% inhibition of Clk4 binding to PGC1α. Using, the same chip and experimental setup, in Figure 5F, we determined that Clk1 binding to PGC1α was only inhibited slightly over 10%. These results indicate that kinase activity and not direct inhibition is the mode by which TG003 modulates Clk1 inhibition of PGC1α.

In Figure 5G, we present select kinases from a 370 kinase panel screen of TG003 inhibition of kinase activity. Kinases provided in Figure 5G show reasonable specificity of TG003 in Clk kinase activity inhibition. Notably, TG003 showed greater than 50% inhibition for Clk1 across the entire kinase panel with the notable exception of approximately 60% inhibition for STK39/SLTK3 and DRYK 1,2,3,4.

## 4 DISCUSSION

In mammals, three main types of adipose depots are classified by their appearance: white, WAT, brown, BAT, and beige, bAT adipose tissues. There are numerous models proposed for demonstrating the transformation of adipocyte precursor cells into beige adipocytes. Brown adipocytes are usually smaller than white and beige adipocytes and contain more mitochondria and multiple small lipid droplets, and are commonly thought to be derived from a different lineage of Myf5‐pre‐adipocytes.^39^ Beige adipocytes contain more mitochondria and smaller lipid droplets.^32^ Our results indicate that inhibition of Clk1, using TG003 during terminal differentiation of 3T3‐L1 adipocytes results in decreased lipid droplet size, decreased cell size and increased mitochondrial number which is consistent with the morphology of beige adipocytes.

Beige adipocytes have a negative impact on obesity due to their expression of specific markers including PPARγ, UCP1 and PGC1α.^7^ Beige adipose cells arise from de novo differentiated adipocytes during cold exposure.^40^, ^41^, ^42^, ^43^ They also develop in mice without prolactin receptors.^44^ Factors that induce angiogenesis and UCP1 thermogenesis regulate WAT browning such as PDGF‐CC.^45^ Dietary factors and phytochemicals such as capsaicin, resveratrol, curcumin, green tea, berberine, fish oil and vitamin A metabolites also function to induce beige adipocytes.^28^ In mice that are globally deficient in PKCβII, there is resistance to DIO.^37^ These mice have adipocytes within subcutaneous WAT that are beige‐like. In our study, the treatment of de novo differentiated adipocytes with TG003, a Clk1/4 inhibitor that also depletes PKCβII protein, resulted in the induction of thermogenic proteins such as UCP1 and PGC1α, markers of bAT transformation.

Mitochondria in adipose tissue contribute to lipolysis and lipogenesis.^46^ Tissue specific functions of mitochondria in white fat are less characterized than in brown fat, but their role in orchestrating metabolic homeostasis and weight control in now accepted.^30^, ^47^ Our finding that UCP1 is highly expressed in TG003‐treated cells is coupled with cells acquiring the features of beige‐like fat.^39^ The finding that 3T3‐L1 pre‐adipocytes utilize Clk 1 kinase for differentiation was demonstrated when we used a Clk1 mutated on each putative Akt phosphorylation sites and showed that all mutations blocked adipognensis.^10^ In our studies, we used siRNA for Clk1, 2 and 4 to demonstrate that Clk1 depletion enhanced UCP1 expression similar to that of TG003 treatment. Thus, we hypothesize that Clk1 and its downstream target for splicing, PKCβII, are involved in the transition from white to beige adipocytes. To further test this, we used the PKCβII specific inhibitor, CGP53353, to show similar reductions in mitochondrial expansion, depletion of lipid stores and reduction of cell size. As mentioned above, PKCβ knockout mice where all alternatively spliced isoforms are depleted, demonstrate the presence of beige‐like cells in their adipose depots.^48^, ^49^ Moreover, PKCβ has been suggested to be a target for therapy to prevent weight gain from long‐term atypical antipsychotic treatment with clozapine.^50^ Clozapine‐induced weight gain was treated with a ruboxistaurine in a preclinical model. Here, PKCβ (without distinction between PKCβI or βII splice variant) was found to cause weight gain during treatment with antipsychotics.^51^

The use of compounds that modulate Clk as agents to prevent diet‐induced obesity (DIO) was as shown earlier where treatment of mice with TG003 was shown to decrease lipid accumulation and result in weight loss with 30–100 mg/kg IP.^52^ TG003 had no effect in chow fed animals, but body weight of DIO mice was reduced by 50% in a course of 23 days. The body temperature of TG003‐treated animals was lower than DIO controls by 1.5° C suggesting that the adipocytes did not generate heat. TG003 was thought to block Clk2 phosphorylation of PGC1α in response to insulin. The authors showed PGC1α and SIRT1 phosphorylation stimulated by insulin was blocked by TG003 and LY294002 (a PI3 kinase inhibitor). Since dosing of animals exceeded the IC_50_ for Clk1/4, Clk2 would also have been blocked. The deposition of TG003 in adipose tissue and the morphology of adipocytes were, however, not assessed in those studies.

We show here that TG003 inhibition of Clk1 in 3T3‐L1 adipocytes resulted in blocking a unique insulin signalling pathway that results in a branch point favouring a new cell phenotype, the beige adipocyte. Our studies with recombinant Clk1 indicated that TG003 and PGC1α both bind to the kinase. TG003 was more specific for Clk1, and siRNA indicated that Clk1 was the cellular kinase responsible for lipid droplet reduction, cell size reduction and mitochondrial biogenesis accompanied with increased proton leak.

We found TG003 highly specific for Clk1 inhibition across a panel of 370 kinases increasing the strength of our hypothesis of Clk1 inhibition for switching a cell program from white to beige‐like adipocytes. The further identification of substrates involved in Clk action that result in increases of UCP1 and PGC1α will enhance the development of drugs that modulate adipocyte function and revise intervention strategies aimed at obesity management. Our study gives insight into the modulatory role of Clk1 kinase inhibition on the de novo differentiation of 3T3‐L1 pre‐adipocytes into beige‐like adipocytes.

## AUTHOR CONTRIBUTIONS

**Achintya Patel:** Data curation (equal); Formal analysis (equal); Investigation (equal); Methodology (equal); Writing – original draft. **Tradd Dobbins:** Data curation (equal); Investigation (equal); Methodology (equal). **Xiaoyuan Kong:** Data curation (supporting); Formal analysis (supporting); Methodology (supporting); Supervision (supporting); Validation (supporting); Visualization (supporting); Writing – original draft (supporting). **Rehka Patel:** Conceptualization (supporting); Data curation (supporting); Methodology (supporting); Validation (supporting). **Gay Carter:** Data curation (supporting); Formal analysis (supporting). **Linette Harding:** Data curation (equal); Methodology (supporting); Software (supporting); Validation (supporting); Visualization (supporting). **Robert P. Sparks:** Data curation (supporting); Methodology (lead); Software (equal); Visualization (supporting); Writing – review & editing (equal). **Niketa A. Patel:** Conceptualization (supporting); Formal analysis (supporting); Funding acquisition (supporting); Methodology (equal); Supervision (supporting); Validation (supporting); Writing – original draft (supporting). **Denisse Cooper:** Conceptualization (lead); Formal analysis (lead); Funding acquisition (lead); Investigation (lead); Project administration (equal); Visualization (lead); Writing – original draft (lead); Writing – review & editing (lead).

## CONFLICT OF INTEREST

The authors have nothing to disclose.
